# The association between diet quality, plant-based diets, systemic inflammation, and mortality risk: findings from NHANES

**DOI:** 10.1007/s00394-023-03191-z

**Published:** 2023-06-22

**Authors:** Yoko Brigitte Wang, Amanda J. Page, Tiffany K. Gill, Yohannes Adama Melaku

**Affiliations:** 1 Vagal Afferent Research Group, School of Biomedicine, University of Adelaide, Adelaide, SA Australia; 2 Nutrition, Diabetes & Gut Health, Lifelong Health Theme, South Australian Health and Medical Research Institute (SAHMRI), Adelaide, SA Australia; 3 Adelaide Medical School, University of Adelaide, Adelaide, SA Australia; 4 FHMRI Sleep Health, College of Medicine and Public Health, Flinders University, Bedford Park, SA Australia; 5 Cancer Epidemiology Division, Cancer Council Victoria, Melbourne, VIC Australia; 6 Department of Cellular and Integrative Physiology, Long School of Medicine, UT Health Science Center at San Antonio, San Antonio, TX 78229 USA

**Keywords:** Inflammation, C-reactive protein, Obesity, Healthy Eating Index, Pro-vegetarian diet, Plant-based dietary index

## Abstract

**Purpose:**

To our knowledge, no studies have examined the association of diet quality and plant-based diets (PBD) with inflammatory-related mortality in obesity. Therefore, this study aimed to determine the joint associations of Healthy Eating Index-2015 (HEI-2015), plant-based dietary index (PDI), healthy PDI (hPDI), unhealthy PDI (uPDI), pro-vegetarian dietary index (PVD), and systemic inflammation with all-cause, cardiovascular disease (CVD), and cancer mortality risks by obesity status.

**Methods:**

Participants from NHANES were included in cross-sectional (*N* = 27,915, cycle 1999–2010, 2015–2018) and longitudinal analysis (*N* = 11,939, cycle 1999–2008). HEI-2015, PDI, hPDI, uPDI, and PVD were constructed based on the 24-h recall dietary interview. The grade of inflammation (low, moderate, and high) was determined based on C-reactive protein (CRP) values and multivariable ordinal logistic regression was used to determine the association. Cox proportional hazard models were used to determine the joint associations of diet and inflammation with mortality.

**Results:**

In the fully adjusted model, HEI-2015 (OR_T3vsT1_ = 0.76, 95% CI 0.69–0.84; *p*-trend =  < 0.001), PDI (OR_T3vsT1_ = 0.83, 95% CI 0.75–0.91; *p* trend =  < 0.001), hPDI (OR_T3vsT1_ = 0.79, 95% CI 0.71–0.88; *p* trend =  < 0.001), and PVD (OR_T3vsT1_ = 0.85, 95% CI 0.75–0.97; *p* trend = 0.02) were associated with lower systemic inflammation. In contrast, uPDI was associated with higher systemic inflammation (OR_T3vsT1_ = 1.18, 95% CI 1.06–1.31; p-trend = 0.03). Severe inflammation was associated with a 25% increase in all-cause mortality (OR_T3vsT1_ = 1.25, 95% CI 1.03–1.53, *p* trend = 0.02). No association was found between PDI, hPDI, uPDI, and PVD with mortality. The joint association, between HEI-2015, levels of systemic inflammation, and all-cause, CVD and cancer mortality, was not significant. However, a greater reduction in mortality risk with an increase in HEI-2015 scores was observed in individuals with low and moderate inflammation, especially those with obesity.

**Conclusion:**

Higher scores of HEI-2015 and increased intake of a healthy plant-based diet were associated with lower inflammation, while an unhealthy plant-based diet was associated with higher inflammation. A greater adherence to the 2015 dietary guidelines may reduce the risk of mortality associated with inflammation and may also benefit individuals with obesity who had low and moderate inflammation.

**Supplementary Information:**

The online version contains supplementary material available at 10.1007/s00394-023-03191-z.

## Introduction

More than 50% of global mortality in 2019 was attributed to chronic conditions, such as cardiovascular diseases (CVD), diabetes, and certain types of cancer [1]. Consumption of a healthy diet can reduce the risk of chronic diseases and mortality [2–4]. This beneficial effect is proposed to be partly mediated by the anti-inflammatory properties of diet which reduces systemic inflammation [5–7]. Current studies have focused on independently examining the association between dietary patterns and systemic inflammation or mortality [4, 8]. To our knowledge, there are no studies investigating the joint association of these factors together. Furthermore, less emphasis was given to the level of inflammation and obesity status, despite the fact they are pivotal predictors of chronic disease risk [9] and potentially mediate the diet–mortality relationship [10]. Therefore, evidence on whether a healthy diet can eliminate mortality risk in different levels of inflammation, and when obesity is present, is warranted.

Adherence to a high diet quality is associated with lower inflammation and mortality risk [8, 11]. In the US, diet quality is often measured using the Healthy Eating Index (HEI), an energy density-based score which assesses the adherence of the US population to the Dietary Guidelines for Americans (DGA) [12]. HEI-2015 was recently developed to reflect the 2015–2020 DGA. It differs from HEI-2010 in the allocation of legumes to all vegetable and protein food components, and inclusion of saturated fats and empty sugars, replacing empty calories, as food group components in the index [12]. With the previous HEI versions, the association between diet quality and inflammatory biomarkers was inconsistent, although it was accepted that high diet quality was associated with lower risk of all-cause, CVD, and cancer mortality [4, 8]. However, evidence for this reduction is scarce when using the HEI-2015.

A global shift toward eating plant-based diets (PBD) has been increasingly promoted given the inverse association with CVD, cancer, type 2 diabetes, and other cardiometabolic risks [13–15], as well as their environmental sustainability impact [16]. Despite this, the previous evidence on PBD mainly focused on the vegetarian diet which exclusively includes plant-based diet and omits animal-sourced food, with no differentiation of PBD quality. Two dietary indices were developed to assess the intake of PBD, a pro-vegetarian diet index (PVD) [17], and plant-based dietary index (PDI) [18]. PVD and PDI are distinct since they include different numbers of food groups into the scoring, 13 and 18 groups, respectively [17, 18]. PVD does not include tea and coffee, sugar sweetened beverages, sweets and desserts, salty food group miscellaneous animal foods [17]. PDI further segregates PBD into healthy (hPDI) and unhealthy PDI (uPDI), allowing assessment of PBD quality [18]. To date, only a few studies have examined PDI and PVD and they showed inconsistency in the association between PBD with inflammation or mortality risks [19–21].

To our knowledge, no studies have examined the joint association between diet quality, PBD, and inflammation with all-cause, CVD, and cancer mortality risks. Therefore, this study aimed to determine: (1) the association between HEI-2015, PDI, hPDI, uPDI, and PVD with C-reactive protein (CRP) and mortality risks; and (2) the joint associations between these indices and systemic inflammation with all-cause, CVD and cancer mortality risks. We hypothesized that adherence to a high diet quality or increased consumption of a PBD is associated with reduced inflammation, and mitigation of inflammatory-associated mortality risks in obesity.

## Materials and methods

This study is reported based on STROBE-nut guidelines (Supplementary Table 1).

### Study design and population

**Fig. 1:**
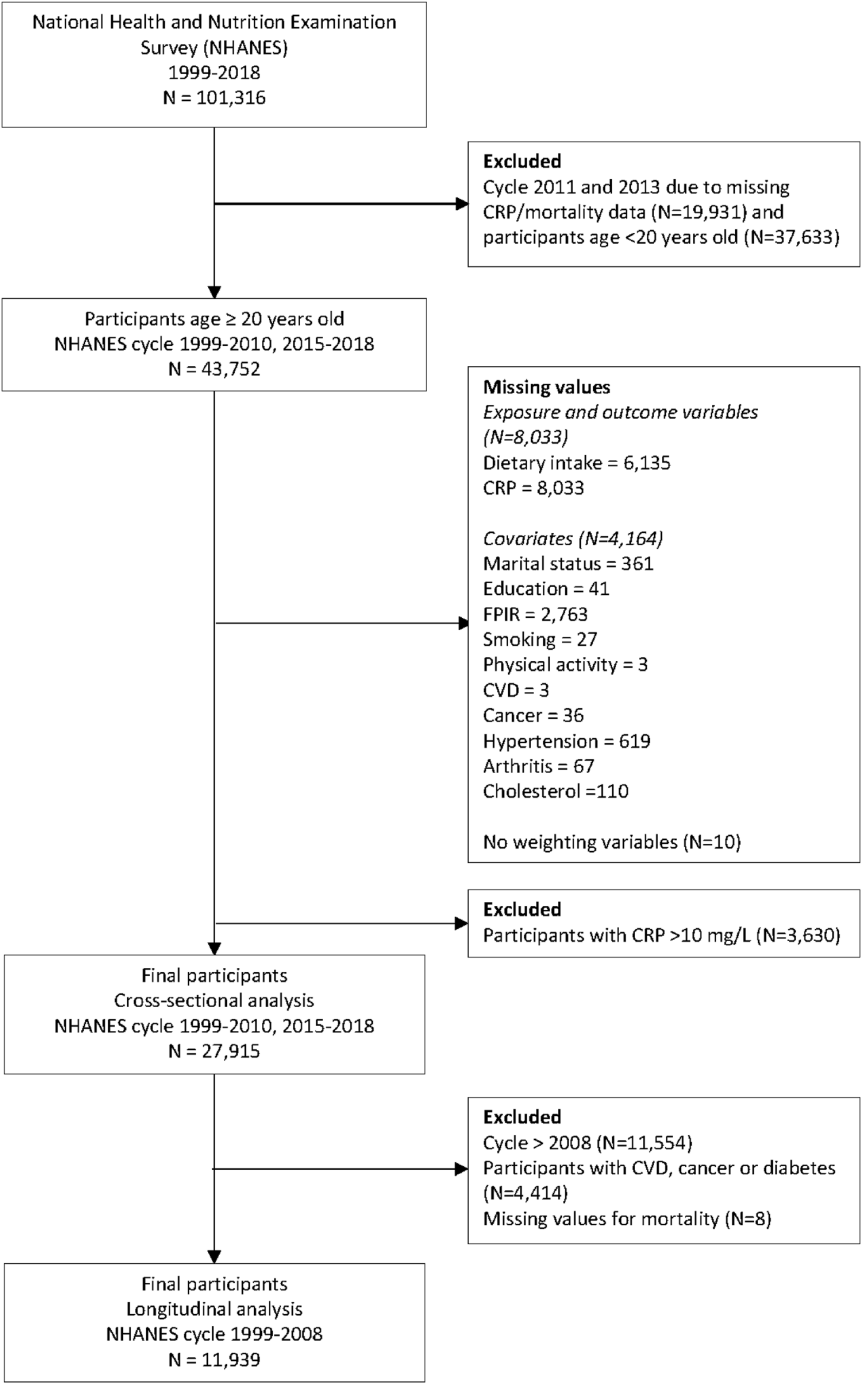
Flowchart of the study design. CRP C-reactive protein, *FPIR* family poverty to income ratio, *BMI* body mass index, *CVD* cardiovascular diseases

This study used the publicly available data from the National Health and Nutrition Examination Survey (NHANES) [22] in the United States. The characteristics and design of the study have been described in detail previously [23]. Participants with missing data and those with CRP levels > 10 mg/L (indicating acute inflammation) [9, 24] were excluded from the study. A total of 27,915 participants from NHANES cycle 1999–2010 and 2015–2018 were included in the cross-sectional analysis to examine the association between HEI-2015, PDI, hPDI, uPDI, and PVD with the grade of inflammation **(**Fig. 1). A total of 11,939 participants from NHANES 1999–2008 were included in the longitudinal analysis to examine the association between HEI-2015, PDI, hPDI, uPDI, PVD, inflammation with all-cause, CVD, and cancer mortality risks. Participants with missing values for exposure and outcome variables, as well as covariates were excluded from the analysis.

### Dietary intake assessment and analysis

Assessment of dietary intake data in NHANES has been described previously [23]. In brief, a repeated 24-h recall interview was used to collect the data. In cycle 1999–2002, one 24-h dietary recall was conducted in-person in the NHANES Mobile Examination Center. From cycle 2003, a second 24-h recall via a telephone interview was conducted approximately 3–10 days after, in addition to the first in-person recall. This study only used the first-day 24-h dietary recall data for several reasons. First, the methods used to obtain dietary intake data were different between days with face-to-face meeting for the first 24-h dietary recall followed by a telephone interview for the remaining dietary data. Second, to facilitate comparison with previous evidence, using the first-day dietary data, we chose to use the face-to-face dietary data. Finally, there was a significant number of missing observations for the second day dietary data. Macronutrient and micronutrient components of food were determined based on the U.S. Department of Agriculture (USDA) Food and Nutrient Database for Dietary Studies 1999–2008 [25]. Food and beverages were classified into 37 components of USDA Food Patterns by linking participant data to the USDA Food Patterns Equivalents Database [25]. The dietary sampling weights were used to account for the complex study design, missing dietary data, and post-stratification.

### Healthy eating index

The overall diet quality of the population was assessed using the HEI-2015 score which reflects adherence to the 2015–2020 DGA [12, 26]. The HEI-2015 includes 13 food and nutrient groups, encompassing 9 adequacy (total fruit, whole fruit, total vegetables, greens and beans, whole grains, dairy, total protein foods, seafood and plant proteins, and fatty acids) and 4 moderation components (refined grains, sodium, added sugars, and saturated fats). The HEI-2015 assesses densities (per calorie) of consumed food groups and nutrients rather than absolute amounts and does not account for nutrients from dietary supplements. Scoring of HEI-2015 is provided in Supplementary Table 2. Briefly, a score of 0–5 was given for total fruits, whole fruits, total vegetables, greens and beans, total protein foods, seafood and plant proteins. A score of 0–10 was allocated to whole grains, dairy, fatty acids, and all moderation components. Scores were given based on energy density (excluding fatty acids) per 1000 cal, with a maximum total score of 100. HEI scores > 80 indicate a good quality diet, a score ranging from 51 to 80 indicates a diet that needs improvement, and a score < 51 reflects poor diet quality [27].

### Pro-vegetarian diet index

We constructed PVD using a method described previously [17]. In brief, it involves consumption of seven plant-sourced food groups (fruit, vegetables, nuts, cereals, legumes, olive oil, and potatoes) and five animal-sourced food groups (added animal fats, eggs, fish, dairy products, meats and meat products) (Supplementary Table 3). Participants consumption of those 12 food groups were divided into deciles and each decile was given a score 1–10. For each participant, positive scores were given to plant-sourced food groups while reverse scores were given to animal-sourced food groups. Scores of an individual from the 12 food groups were summed to obtain the indices. The scores for PVD range from 12 to 120 [17].

### Plant-based dietary indices

PDI, hPDI, and uPDI were constructed following a previously described method [18]. In brief, food items were condensed into 18 food groups based on nutrient and culinary similarities and classified under 3 larger categories: animal-based foods, healthy and less healthy plant-based foods (Supplementary Table 3). Healthy and less healthy plant-based foods were separated based on the existing knowledge on the association between plant food components with intermediate conditions and health outcomes [18]. Margarine was excluded from the indices as the fatty acids content has changed from high levels of trans fat to unsaturated fats [18]. Alcoholic beverages were also excluded from the indices given their unclear association with different health outcomes. However, alcohol was adjusted for in the analysis [18].

Food groups were divided into deciles and each decile was given a score 1–10. For PDI, a score of 10 was given to participants for each plant food group, if they were above the highest decile of consumption, a score of 9 if they were above the second highest decile but below the highest decile, and so on. A score of 1 was allocated for consumption below the lowest decile. For each animal food group, reverse scores were given to the participants. For hPDI, positive scores were allocated to healthy plant food groups while reverse scores were given to less healthy plant and animal food groups. Conversely, positive scores were given to less healthy plant food groups, and reverse scores to healthy plant and animal food groups, for uPDI. Scores for the 18 food groups were summed for each participant to obtain the indices. The possible score range was 18 to 180.

### Measurement of CRP, mortality, and other covariates

The CRP (high sensitivity) values were examined using an immunoturbidimetric system from the collected blood samples. The detailed method for each cycle has been described previously [28]. For the cross-sectional analysis, the CRP values were categorized into low (< 3 mg/L) and severe (≥ 3 mg/L) [29]. For the longitudinal analysis, the CRP values were categorized into low (< 1 mg/L), moderate (1–3 mg/L), and high (> 3 mg/L) inflammation [9].

Ascertainment of mortality for the included participants were obtained from National Center for Health Statistics Public-Use Linked Mortality Files with a probabilistic matching algorithm to the National Death Index [30]. We considered the mortality data up to 2015 for participants in the NHANES cycle 1999–2008 to allow sufficient lag time to determine mortality rates [31], with a mortality status equal to 0 considered to be alive through to the end of 2015.

We selected covariates based on previous literature and summarized in a directed acyclic diagram **(**Supplementary Fig. 1). Those covariates include sociodemographic factors (age, sex, race, marital status, education, family poverty to income ratio), behavioral factors (smoking, physical activity, alcohol intake), chronic conditions (CVD, cancer, arthritis, diabetes, hypertension, cholesterol), and body mass index (BMI). Measurement of the covariates has been described in the NHANES survey methods and analytics guidelines [23] and NHANES Laboratory Data [28]. A summary of assessment methods and type of the covariates data is provided in Supplementary Table 4.

### Statistical analyses

A complete case analysis was used. HEI-2015, PDI, hPDI, uPDI, and PVD were categorized into tertiles based on participant intake scores. Categorization was undertaken to facilitate interpretation of association estimates in relevance to clinical settings and public health practice and to allow comparison with other studies.

Descriptive analysis of covariates was performed across tertiles of dietary indices. For continuous and normally distributed variables, mean, median and standard deviation were calculated. For categorical variables, proportions were used. Significant differences across dietary indices tertiles were determined using ANOVA and Chi-square tests. Multivariable logistic regression was used to determine the odds ratio of the association between dietary measures and the grade of inflammation. Cox proportional hazard models were used to examine the association between the dietary indices and all-cause, CVD and cancer mortality.

Four models were developed for the cross-sectional and longitudinal analysis. Model one was adjusted for sociodemographic factors. Model two was additionally adjusted for behavioral factors. Model three was additionally adjusted for chronic conditions. Model four was additionally adjusted for BMI and total energy intake (PBD and PVD models only). The p value for trend was determined using tertiles as a continuous variable.

We developed additional Cox proportional hazard models, with exposure variables that combine dietary measure tertiles and inflammation levels to determine the joint association of HEI-2015, systemic inflammation and mortality. We derived a joint variable that combined the HEI-2015 score and inflammation levels. The participants in the highest tertile of HEI-2015, the low inflammation group, served as the reference.

Subgroup analysis based on obesity status was performed in both cross-sectional analysis on the association between HEI-2015, PDI, hPDI, uPDI, and PVD with systemic inflammation, and the joint association between HEI-2015, systemic inflammation, and all-cause mortality. Obesity status was determined based on BMI following the World Health Organization definition, with BMI ≥ 30 km/m^2^ defined as obesity and BMI < 30 kg/m^2^ defined as non-obesity [32]. Stratification by obesity status was determined based on a theoretical justification.

The statistical analyses were performed with STATA/SE version 17 (Stata, StataCorp LP, College Station, TX, USA). Data visualization was performed using RStudio version 2022.02.3 [33] and R package *ggplot2* [24].

## Results

### Characteristic of participants

Characteristics of participants for the cross-sectional analysis are presented in Table 1 and Supplementary Table 5. Participants in higher tertiles of the HEI-2015, PDI, hPDI, and PVD were likely to be older, married or living with a partner, more physically active, non-/ex-smoker, and had a higher level of education. In contrast, participants in the higher tertiles of uPDI were likely to be younger, had a lower physical activity level, and had a BMI ≥ 30 kg/m^2^. HEI-2015 scores of the participants ranged between 8 and 100 with a mean score of 50.4, suggesting a poor diet quality [27]. The mean follow-up year was 11.2 ± 3.2, 1149 deaths were attributable to all-causes, 222 deaths were caused by CVD, 263 deaths were caused by cancer, and the overall mortality rate was 5.3 per 1000 person-years.

**Table 1:** Descriptive analysis of participant characteristics based on tertiles of HEI-2015, PDI and PVD in NHANES (*N* = 27,915)

Covariates	Overall	HEI-2015			*p* trend	PDI			*p* trend	PVD			*p* trend
		T1	T2	T3		T1	T2	T3		T1	T2	T3	
Age (years) (median, IQR)	49 (35, 65)	41 (30, 54)	46 (33, 58)	52 (38, 65)	< 0.001	42 (30, 56)	46 (34, 60)	49 (36, 61)	< 0.001	45 (32, 58)	46 (32, 60)	47 (34, 60)	< 0.001
Sex (*n*%)													
Male	50.6	54.1	51.1	46.4	< 0.001	52.7	49.5	49.5	0.001	53.7	49.5	48.8	< 0.001
Female	49.4	45.9	48.9	53.6		47.3	50.5	50.5		46.3	50.5	51.2	
Race (*n*%)													
Mexican American	7.8	8.1	8.4	6.8	< 0.001	7.7	8.1	7.5	< 0.001	7.3	7.7	8.2	< 0.001
Other Hispanic	4.8	4.4	5.5	4.7		5.3	5.1	4.1		4.8	5	4.8	
Non-Hispanic White	71.5	72.5	69.1	72.8		68.2	71.7	74.6		69.5	71.6	73.3	
Non-Hispanic Black	9.6	10.1	10.4	8.2		12.6	9.4	6.9		12.8	9.4	6.8	
Other Race including multi-racial	6.3	4.8	6.6	7.5		6.2	5.7	6.9		5.6	6.3	6.9	
Marital status (*n*%)													
Married/living with partner	64.2	61.4	64.1	67.2	< 0.001	58.8	64.5	69.2	< 0.001	59.5	64.7	68	< 0.001
Widowed	5.9	4.4	5.8	7.8		5.5	6.4	5.9		6.1	5.7	6	
Divorced	10.0	10.5	10.3	9.3		11.4	10	8.8		12.4	9.6	8.3	
Separated	2.4	2.9	2.4	2		2.6	2.5	2.2		2.7	2.4	2.1	
Never married	17.4	20.9	17.5	13.7		21.8	16.6	13.9		19.3	17.6	15.5	
Education (*n*%)													
Less than high school	16.1	19	16.7	12.3	< 0.001	19.3	19.3	16.1	< 0.001	19.2	19.2	15.9	< 0.001
High school diploma (including GED)	24.9	29.7	24.9	19.7		27.5	27.5	24.8		27	27	25.9	
More than high school	59.1	51.3	58.3	68		53.2	53.2	59.1		53.8	53.8	58.2	
FPIR (median, IQR)	3.1 (1.6, 5)	2.7 (1.3, 4.7)	3.1 (1.6, 5)	3.6 (1.9, 5)	< 0.001	2.7 (1.4, 4.7)	3.1 (1.6, 5)	3.6 (1.9, 5)	< 0.001	2.8 (1.4, 4.8)	3.1 (1.6, 5)	3.5 (1.8, 5)	< 0.001
Smoking (*n*%)													
Never smoked	53.0	47.3	53	58.8	< 0.001	49.8	53.5	55.6	< 0.001	47.9	53.1	57.5	< 0.001
Ex-smoker	25.3	22.1	24.4	29.6		23	25	28		24.4	25.2	26.4	
Smoker	21.7	30.6	22.6	11.5		27.2	21.5	16.4		27.7	21.7	16.1	
Physical activity level (*n*%)													
Low	35.4	38.2	37	30.9	< 0.001	36.3	37.1	32.8	< 0.001	38	35.7	32.7	< 0.001
Moderate	12.8	12.4	13	13.2		12.4	12.1	14		12.1	13	13.4	
High	51.8	49.5	50	55.9		51.3	50.8	53.2		49.9	51.4	53.9	
Alcohol intake (gm) (median, IQR)	0 (0, 3.7)	0 (0, 0)	0 (0, 13.2)	0 (0, 11.2)	< 0.001	0 (0, 13)	0 (0, 0.8)	0 (0, 0.2)	< 0.001	0 (0, 11.2)	0 (0, 1.6)	0 (0, 0.5)	< 0.001
CVD (*n*%)													
No	91.5	92.4	92	90.2	< 0.001	92.3	90.8	91.5	0.01	91	91.6	92	0.16
Yes	8.5	7.6	8	9.8		7.7	9.2	8.5		9	8.4	8	
Cancer (*n*%)													
No	90.7	92	91.4	88.5	< 0.001	91.6	90.9	89.5	0.001	91.2	90.9	89.9	0.08
Yes	9.3	8	8.6	11.5		8.4	9.1	10.5		8.8	9.1	10.1	
Hypertension (*n*%)													
No	68.7	71.8	69.8	64.3	< 0.001	69.7	68.5	68	0.23	67.6	68.8	69.7	0.09
Yes	31.3	28.2	30.2	35.7		30.3	31.5	32		32.4	31.2	30.3	
Arthritis (*n*%)													
No	75.4	77.5	75.7	72.9	< 0.001	77.6	74.6	73.9	< 0.001	75.1	74.2	76.9	0.01
Yes	24.6	22.5	24.3	27.1		22.4	25.4	26.1		24.9	25.8	23.1	
Diabetes (*n*%)													
No	88.7	90.2	89	86.7	< 0.001	88.7	87.8	89.6	0.01	87.7	88.5	89.8	0.01
Yes	11.3	9.8	11	13.3		11.3	12.2	10.4		12.3	11.5	10.2	
Cholesterol (mean, SD)	5.1 (1.1)	5.1 (1.1)	5.1 (1.1)	5.1 (1.1)	0.02	5.1 (1.1)	5.1 (1.1)	5.1 (1.1)	0.09	5.1 (1.1)	5.1 (1.1)	5.1 (1.1)	0.99
BMI (mean, SD)	28.3 (6.1)	28.6 (6.2)	28.4 (6.2)	27.6 (5.8)	< 0.001	28.7 (6.4)	28.3 (6.1)	27.7 (5.8)	< 0.001	28.8 (6.5)	28.3 (6.1)	27.6 (5.7)	< 0.001
Obesity (*n*%)													
No	67.0	63.2	66.2	71.8	< 0.001	63.9	66.4	70.7	< 0.001	62.9	66.4	71.4	< 0.001
Yes	33.0	36.8	33.8	28.2		36.1	33.6	29.3		37.1	33.6	28.6	
CRP (*n*%)													
< 1.0 mg/L	34.4	31.6	33.9	37.9	< 0.001	30.7	34.2	38.3	< 0.001	30.2	33.7	39	< 0.001
1.0–3.0 mg/L	37.5	36.8	38.2	37.6		38	37.9	36.7		38.2	38.3	36.1	
> 3.0 mg/L	28.1	31.6	27.9	24.5		31.3	27.9	25		31.7	28	24.9	

*HEI-2015* Healthy Eating Index-2015, *PDI* Plant-based dietary index, *PVD* pro-vegetarian diet, *CRP* C-reactive protein, *FPIR* family poverty income ratio, CVD cardiovascular diseases, *BMI* body mass index

ANOVA was used for quantitative variables and Pearson’s Chi-square test was used for categorical variables

### Association between dietary indices and inflammation

**Fig. 2:**
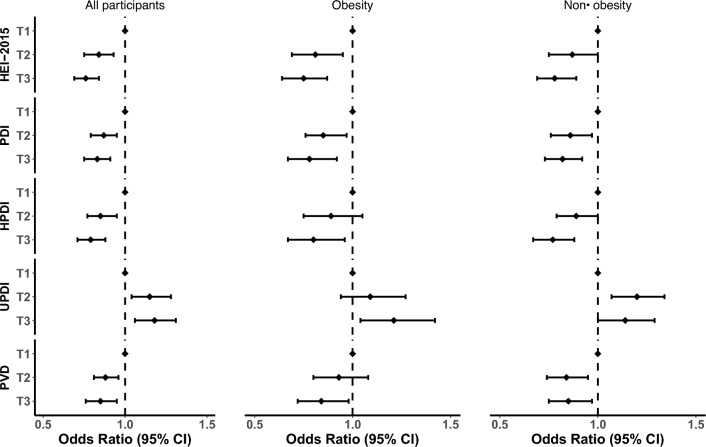
Odds ratio of the association between different dietary indices and systemic inflammation in a fully adjusted model. *HEI-2015* Healthy Eating Index-2015, *PDI* plant-based dietary index, *HPDI* healthy plant-based dietary index, *UPDI* unhealthy plant-based dietary index, *PVD* pro-vegetarian dietary pattern, *CI* confidence interval. Total participants (*N* = 27,915), individuals without obesity (*N* = 18,454), individuals with obesity (*N* = 9461)

The odds ratio of the association between dietary indices and inflammation in the fully adjusted model is presented in Fig. 2** (**details for the other models are provided in Supplementary Table 6–8). HEI-2015 (OR_T3vsT1_ = 0.76, 95% CI 0.69–0.84; *p* trend =  < 0.001), PDI (OR_T3vsT1_ = 0.83, 95% CI 0.75–0.91; *p* trend =  < 0.001), hPDI (OR_T3vsT1_ = 0.79, 95% CI 0.71–0.88; p trend =  < 0.001), and PVD (OR_T3vsT1_ = 0.85, 95% CI 0.75–0.97; *p* trend = 0.02) were associated with lower systemic inflammation in the fully adjusted model, in all participants. In contrast, uPDI was associated with higher systemic inflammation (OR_T3vsT1_ = 1.18, 95% CI 1.06–1.31; *p* trend = 0.03). The association between all dietary indices and systemic inflammation remained strong in participants with or without obesity with no marked changes in the effect size, except for uPDI in participants without obesity (OR_T3vsT1_ = 1.14, 95% CI 1.00–1.29; *p*-trend = 0.03).

### Joint association between HEI-2015 and mortality

**Fig. 3:**
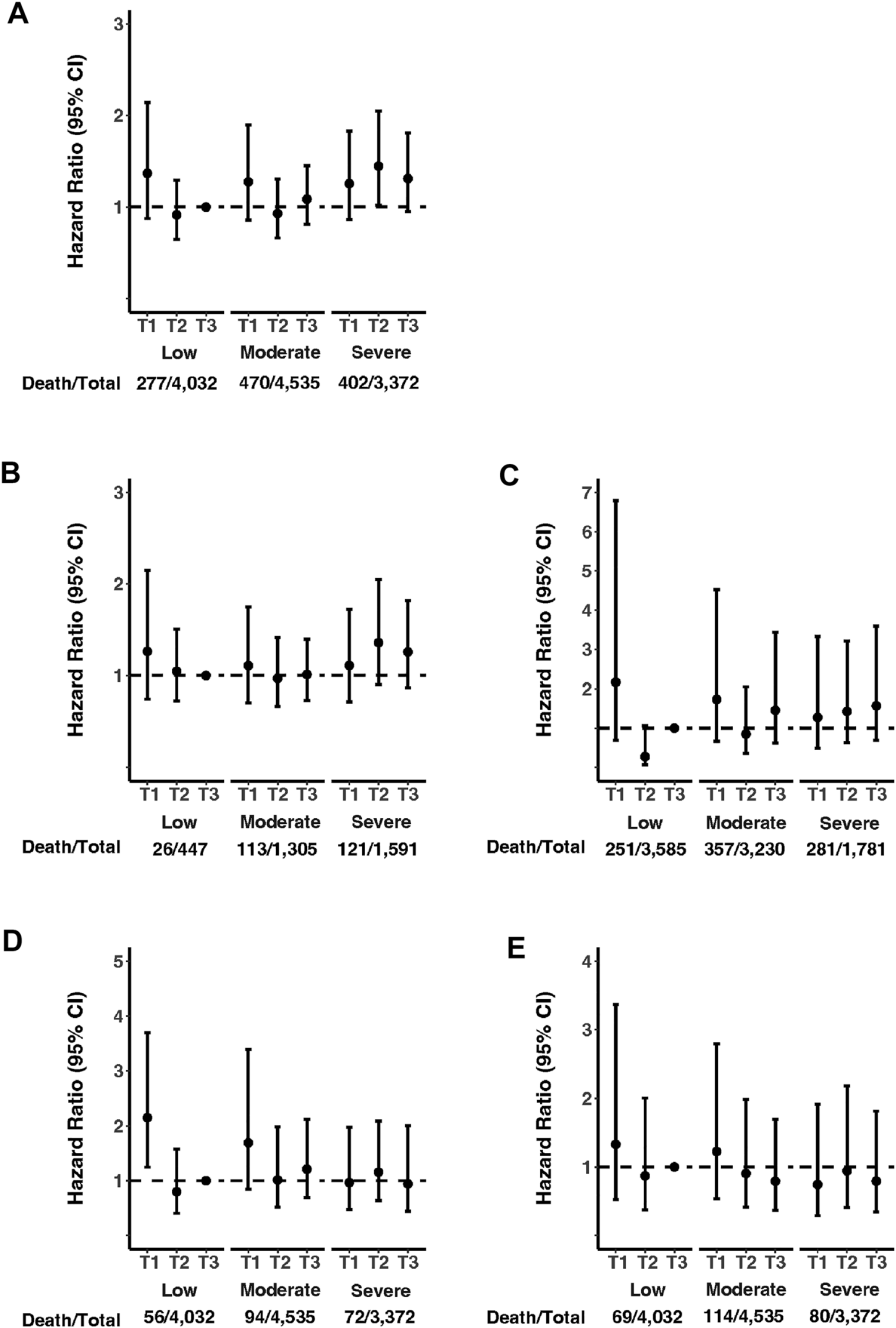
Joint association between HEI-2015, systemic inflammation and **A** all-cause mortality risk in the total population; **B** all-cause mortality risk in the non-obesity group; **C** all-cause mortality risk in the obesity group; D) CVD mortality risk; and **E** cancer mortality risk. Total participants (*N* = 11,939), individuals without obesity (*N* = 8596), individuals with obesity (*N* = 3343). *T1, T2, T3* HEI-2015 categories, *low* moderate, *severe* hs-CRP categories. Number of death/total participants is presented for hs-CRP categories. *CI* confidence interval

The association between HEI-2015, CRP, and mortality risks is presented in Table 2**.** In the fully adjusted model, the highest tertile of CRP was associated with all-cause mortality risk in all participants (OR_T3vsT1_ = 1.25, 95% CI 1.03–1.53, *p* trend = 0.02). The highest tertile of HEI-2015 was also associated with reduced all-cause mortality risk in all participants. Association between severe inflammation and all-cause mortality was stronger in people with obesity compared to those without obesity. HEI-2015 was associated with reduced CVD and cancer mortality. No association was observed between CRP with CVD or cancer-associated mortality risk. We also explored the association between PBD indices and PVD with mortality; however, no association was found in the fully adjusted model for all participants (Supplementary Table 9). The joint associations between HEI-2015 and the grade of inflammation with all-cause, CVD, and cancer mortality are presented in Fig. 3 and Supplementary Table 10. No association was found for the joint associations, except for all-cause mortality risk in participants with severe inflammation in the second tertile of HEI-2015 (HR_T3vsT1_ = 1.45, 95% CI 1.02–2.05) of the fully adjusted model. A greater reduction of mortality risk with an increase of HEI-2015 scores (tertile 1 to tertile 2) was observed in individuals with low and moderate inflammation. When comparing people with and without obesity, adherence to higher scores of HEI-2015 was not associated with decreased all-cause mortality in participants with obesity who had severe inflammation.

**Table 2:** Hazard Ratios of all-cause, CVD, and cancer mortality risk based on tertiles of HEI-2015 and CRP

	HEI-2015				Inflammation (CRP)			
Model	Hazard ratio (95% confidence interval)			*p* trend	Hazard ratio (95% confidence interval)			*p* trend
	T1	T2	T3		Low	Moderate	Severe	
All cause								
All population								
Death/Total	331/3980	372/3980	446/3979		277/4032	470/4535	402/3372	
Model 1	1.00	0.81 (0.68–0.98)	0.80 (0.64–0.99)	0.05	1.00	1.02 (0.82–1.28)	1.27 (1.05–1.54)	**0.01**
Model 2	1.00	0.82 (0.69–0.99)	0.88 (0.71–1.09)	0.26	1.00	0.98 (0.78–1.23)	1.18 (0.98–1.42)	0.06
Model 3	1.00	0.83 (0.69–1.00)	0.88 (0.71–1.09)	0.25	1.00	0.98 (0.78–1.23)	1.17 (0.97–1.43)	0.08
Model 4	1.00	0.83 (0.69–1.00)	0.87 (0.70–1.08)	0.24	1.00	1.02 (0.81–1.29)	1.25 (1.03–1.53)	**0.02**
Obesity								
Death/Total	89/1228	70/1095	101/1020		26/477	113/1305	121/1591	
Model 1	1.00	0.63 (0.42–0.96)	0.82 (0.58–1.16)	0.30	1.00	1.17 (0.65–2.11)	1.24 (0.70–2.18)	0.43
Model 2	1.00	0.64 (0.42–0.97)	0.91 (0.65–1.28)	0.60	1.00	1.15 (0.64–2.07)	1.21 (0.69–2.13)	0.48
Model 3	1.00	0.66 (0.44–0.99)	0.92 (0.65–1.29)	0.65	1.00	1.17 (0.65–2.11)	1.25 (0.71–2.20)	0.41
Non-obesity								
Death/Total	242/2752	302/2885	345/2959		251/3585	357/3230	281/1781	
Model 1	1.00	0.94 (0.75–1.18)	0.85 (0.65–1.10)	0.21	1.00	1.00 (0.78–1.28)	1.30 (1.04–1.62)	**0.03**
Model 2	1.00	0.95 (0.76–1.19)	0.94 (0.72–1.23)	0.66	1.00	0.95 (0.74–1.23)	1.17 (0.93–1.46)	0.18
Model 3	1.00	0.95 (0.76–1.19)	0.93 (0.72–1.21)	0.60	1.00	0.95 (0.73–1.22)	1.15 (0.91–1.45)	0.27
CVD								
All population								
Death/Total	74/3980	64/3980	84/3979		56/4032	94/4535	72/3372	
Model 1	1.00	0.62 (0.41–0.95)	0.62 (0.42–0.92)	**0.03**	1.00	1.12 (0.80–1.57)	0.93 (0.65–1.35)	0.68
Model 2	1.00	0.63 (0.42–0.97)	0.69 (0.47–1.02)	0.08	1.00	1.03 (0.73–1.47)	0.82 (0.56–1.21)	0.29
Model 3	1.00	0.64 (0.42–0.99)	0.69 (0.47–1.03)	0.08	1.00	1.01 (0.71–1.43)	0.80 (0.54–1.18)	0.23
Model 4	1.00	0.64 (0.42–0.99)	0.69 (0.47–1.02)	0.08	1.00	1.03 (0.70–1.51)	0.82 (0.55–1.22)	0.28
Cancer								
All population								
Death/Total	81/3980	83/3980	99/3979		69/4032	114/4535	80/3372	
Model 1	1.00	0.82 (0.53–1.28)	0.72 (0.44–1.19)	0.20	1.00	0.95 (0.59–1.52)	0.85 (0.51–1.40)	0.51
Model 2	1.00	0.83 (0.54–1.28)	0.78 (0.48–1.28)	0.33	1.00	0.90 (0.56–1.46)	0.77 (0.47–1.27)	0.31
Model 3	1.00	0.82 (0.53–1.28)	0.78 (0.48–1.27)	0.32	1.00	0.91 (0.56–1.49)	0.79 (0.48–1.30)	0.35
Model 4	1.00	0.82 (0.53–1.28)	0.77 (0.48–1.26)	0.31	1.00	0.92 (0.55–1.55)	0.80 (0.47–1.36)	0.41

Hazard ratio from multivariable Cox proportional hazards. *HEI-2015* Healthy Eating Index-2015, *CRP* C-reactive protein, *CVD* cardiovascular diseases. Total participants (*N* = 11,939), non-obesity (*N* = 8596), obesity (*N* = 3343)

Model 1: adjusted for socioeconomic factors (sex, age, race, marital status, education, family poverty to income ratio)

Model 2: additionally adjusted for behavioral factor (PAL, smoking, alcohol intake)

Model 3: additionally adjusted for chronic conditions (CVD, cancer, diabetes, arthritis, cholesterol, hypertension)

Model 4: additionally adjusted for BMI

Bold denoted significant *p* value (< 0.05)

## Discussion

This study is the first, to our knowledge, to examine the joint association of diet quality and inflammation with all-cause, CVD, and cancer mortality, in healthy and people with obesity. We found that higher adherence to HEI-2015, overall/healthy PDI, or PVD was associated with lower inflammation. In the longitudinal analysis, higher HEI-2015 scores were associated with a reduced CVD mortality risk. A greater adherence to the dietary guidelines may mitigate inflammation-associated all-cause, CVD, and cancer mortality risks, particularly for participants with moderate levels of inflammation.

### Diet quality, plant-based diets, and inflammation

Our results relating to diet quality and inflammation are consistent with previous cross-sectional studies using HEI-2015. The Mitchelstown cohort of the Cork and Kerry Diabetes and Health Diseases Study in Ireland reported that levels of inflammatory biomarkers (i.e., CRP, IL-6 and TNF-α) were lower in individuals with higher HEI-2015 scores[34]. A study of NHANES 2005–2010 reported the highest quintile of HEI-2015 was associated with a 17% lower level of hs-CRP in adults [29]. In our study using NHANES 1999–2010 and 2015–2018, we found higher scores of HEI-2015 were associated with a 16–24% reduction in hs-CRP. This finding is also in accordance with evidence using the previous HEI versions [35, 36], although some have reported no association in studies only using female participants [37, 38].

Our study is one among only a few that have explored the association between PBD and inflammation. We found that adherence to a healthy PDI was associated with a 15–21% reduction in hs-CRP. This is in accordance with the Nurse’s Health Study (NHS), where healthy PDIs were associated with a reduction in hs-CRP levels in cross-sectional (13.6%) and longitudinal (17.8%) analysis [39]. In addition, a lower hs-CRP level was associated with an increased hPDI score in Saudi Arabian [40] and American females with overweight/obesity [41]. We also showed that unhealthy PDI were associated with increased inflammation, in line with previous observational studies [39, 40, 42]. Furthermore, this study revealed an inverse association between overall PDI and inflammation which diverged from previous evidence, reporting no association between overall PDI and hs-CRP [39, 40, 42]. This could be due to the small sample size included in the previous studies. In addition, a study of the NHS cohort reported that BMI attenuated the association [39]. Consistent with this observation, our fully adjusted model demonstrated a similar effect of BMI, although it did not affect the direction of association.

We also found that the PVD was associated with a 12–15% reduction in hs-CRP, consistent with the results of the overall PDI. PVD has been associated with a lower risk of metabolic syndromes [17, 43] and cardiovascular-related risks [44] but no study has explored PVD with inflammation as an outcome, particularly in the US population. This result is expected given that PVD and overall PDI share similar food groupings and scoring methods [17, 18]. In support to our findings, a recent systematic review showed that studies using indices that emphasize intake of PBD (e.g., DASH, vegan/vegetarian pattern, HEI-2010, Mediterranean and paleolithic diet) were associated with lower levels of oxidative stress and inflammatory biomarkers, including hs-CRP [45].

When comparing between HEI-2015, overall PDI, and PVD in the association with hs-CRP, we found the association was stronger for HEI-2015, followed by the overall PDI and PVD. This suggests that HEI-2015 could be a better predictor of inflammation. HEI-2015 is constructed based on a combination of food groups and nutrient components [12]. This is different from PDI and PVD where both only consider food groups [17, 18]. In accordance to our findings, this may indicate that including nutrient components in dietary indices is necessary to examine the association between diet and inflammation.

Furthermore, subgroup analysis by obesity status revealed a greater reduction of hs-CRP in individuals with obesity compared to ones without obesity who adhere to high diet quality and overall PBD (HEI-2015:19–25% vs. 13–22%, PDI:15–22% vs 14–18%). Altogether, our cross-sectional results suggest that higher diet quality and higher intake of healthy PBD were associated with lower systemic inflammation with a greater effect in participants with obesity.

### Joint associations of diet and inflammation on mortality

We showed that HEI-2015 and hs-CRP are independent predictors of mortality. Adherence to HEI-2015 was associated with a reduction in CVD mortality. Likewise, HEI-2015 was associated with all-cause and cancer mortality. These findings are in line with several cohort studies in the general US population [46, 47] and Spain [48].

We found no association between higher intake and the quality of PBDs with all-cause mortality. Although this is surprising, given PBDs have been associated with lower risks of CVD, cancer, and type 2 diabetes [49–51]. Previous evidence reporting the association between PBDs and mortality is inconsistent [21, 52, 53]. A study of NHANES III (1988–1994) reported no association between overall PDI and uPDI with all-cause and CVD mortality while a non-linear association was observed between hPDI and lower all-cause mortality risk [21]. Conversely, associations between overall PDI and hPDI with lower all-cause mortality risk, and uPDI with higher all-cause mortality risk, were found in a study using NHANES 1999–2014 data [52]. Our results for PVD also differ from previous studies which reported higher PVD scores with lower all-cause mortality risk in the US [20] and older Spanish [17, 54] population. These may be due to differences in methodological approaches (e.g., variation in dietary intake data used, inaccuracies in cause-of-death information) and the possibility that participants changed their dietary habits during the follow-up time. Further examination is warranted to confirm our findings.

The joint association suggests that a greater adherence to the dietary guidelines may reduce all-cause, CVD, and cancer mortality risk in all grades of inflammation. This protective effect is biologically plausible since a healthier diet, or high diet quality, is characterized by intake of vegetables, fruits, nuts, and legumes, which compose the healthy plant-based foods. These food groups contain anti-inflammatory and antioxidants components (e.g., dietary fibers, vitamins, polyphenols, and unsaturated fatty acids) which reduce inflammation [5–7]. Conversely, poor diet or low diet quality, often characterized by animal-based foods (e.g., red meat, saturated fats) and unhealthy plant-based foods, was associated with increased inflammation, partly due to their pro-inflammatory properties [55, 56]. In obesity, all-cause mortality risk was lower with greater adherence to the dietary guidelines in low and moderate inflammation but not for severe inflammation. This indicates that a diet with anti-inflammatory properties alone might not be effective to reduce mortality risk in the obesity. This is likely due to the tenacious nature of low-grade systemic inflammation in obesity [57].

We also observed a weak or null association in the joint associations. This may be due to the scoring of protein intake in the index. In the last two versions, HEI-2010 [58] and HEI-2015 [47], protein intake is separated into total protein foods and seafood and plant proteins components to indicate consumption preference. However, in the earlier versions, HEI-1995 [59] and HEI-2005 [60], they were allocated to the meat (and beans) component. Thus, this may affect the strength of association. The alternate healthy eating index (AHEI) has been suggested as a better predictor of chronic diseases risk compared to HEI [61, 62]. However, the focus of this study was the association between adherence to the dietary guidelines, inflammation, and mortality. As AHEI may not reflect adherence to the dietary guidelines, the HEI was chosen. In addition, it is also plausible that different CRP cutoff levels used to classify inflammation status could influence the strength of the association. Future studies are warranted to confirm the suitability of HEI and the use of AHEI to assess the joint association between systemic inflammation and mortality.

### Strengths and limitations

The study strengths include the large number of participants, representative of the general US adult population, with a mean of 11.2-year follow-up. The study is one among the few that has used the general adult population data to examine the association between diet quality, PBDs, inflammation, and mortality in prospective cohort design [47, 52, 63, 64]. We also only included participants who completed a face-to-face 24-h dietary recall given there was a significant number of missing observations for the second day dietary data; and to facilitate comparison with previous evidence.

There are some limitations which should be considered. Given dietary intake data were only obtained at baseline with repetitive assessment unavailable with the NHANES data, it is possible that participants changed their diets during the follow-up period which may impact on the associations. The percentage of missing data that were excluded in this study was quite high (e.g., > 10% for hs-CRP values). This might impact on the strength of association. The use of 24-h dietary recall may not fully reflect habitual dietary intake of the participants since it is unable to account day-to-day variation. Furthermore, the grade of inflammation was assessed solely based on hs-CRP levels. Nonetheless, CRP is widely used as a clinical biomarker of inflammation [65], is a predictor of CVD risk [9], and is associated with increased risk of all-cause and cancer mortality [2]. We were not able to determine causality given the nature of observational studies, there are potential residual confounders that may affect the association. In addition, caution should be taken in interpreting results with wide confidence intervals, which could be due to a sample size limitation in subgroups.

## Conclusion

We found that higher scores for HEI-2015 and healthy PDI were associated with lower inflammation, while an unhealthy plant-based diet was associated with higher inflammation. A greater adherence to the dietary guidelines may reduce all-cause, CVD, and cancer mortality risk in all grades of inflammation. Adherence to the dietary guidelines may also benefit individuals with obesity where the inflammatory status falls into the low and moderate range. Our findings support dietary guidelines, recommendations, and health promotion activities that highlight adherence to high diet quality, increased intake of healthy PBDs, and reduced consumption of animal-sourced diets for better health. Importantly, they suggest the need for improving dietary guidelines or recommendations that are more focused on addressing inflammation by (1) emphasizing plant-based diet quality, (2) considering pro/anti-inflammatory nutrient components, and 3) tailoring the recommendations for population subgroups based on obesity and inflammatory status. Further studies that include different inflammatory biomarkers, repeated assessment of dietary intake data, and different populations are also warranted to further examine the benefit of high diet quality and plant-based diet consumption in reducing inflammation and mortality.

## Funding

YBW is supported by an Australia Awards Scholarship from The Department of Foreign Affairs and Trade, The Government of Australia. YAM is supported by a National Health and Medical Research Council of Australia (NHMRC) an Investigator Grant (2009776).

## Supplementary Information

Below is the link to the electronic supplementary material.

Supplementary file1 (DOCX 5580 KB)

## Data Availability

Not applicable.
