# Supplementary material for: The association between diet quality, plant-based diets, systemic inflammation, and mortality risk: findings from NHANES
Source: Eur J Nutr. 2023 Jun 22;62(7):2723–37. doi: 10.1007/s00394-023-03191-z (PMC10468921; doi:10.1007/s00394-023-03191-z)

**Supplementary Table 1.** STROBE-nut report of the study.

| **Item** | **Item nr** | **STROBE recommendations** | **Extension for Nutritional Epidemiology studies (STROBE-nut)** | **Reported on page #** |
| --- | --- | --- | --- | --- |
| **Title and**  **abstract** | 1 | (a) Indicate the study’s design with a commonly used term in the title or the abstract.  (b) Provide in the abstract an informative and balanced summary of what was done and what was found. | **nut-1** State the dietary/nutritional assessment method(s) used in the title, abstract, or keywords. | 1, 3 |
| **Introduction** |  |  |  |  |
| Background rationale | 2 | Explain the scientific background and rationale for the investigation being reported. |  | 5-6 |
| Objectives | 3 | State specific objectives, including any pre-specified hypotheses. |  | 6 |
| **Methods** |  |  |  |  |
| Study design | 4 | Present key elements of study design early in the paper. |  | 6 |
| Settings | 5 | Describe the setting, locations, and relevant dates, including periods of recruitment, exposure, follow-up, and data collection. | **nut-5** Describe any characteristics of the study settings that might affect the dietary intake or nutritional status of the participants, if applicable. | 6 |
| Participants | 6 | a) Cohort study—Give the eligibility criteria, and the sources and methods of selection of participants. Describe methods of follow-up.  Case-control study—Give the eligibility criteria, and the sources and methods of case ascertainment and control selection. Give the rationale for the choice of cases and controls.  Cross-sectional study—Give the eligibility criteria, and the sources and methods of selection of participants.  (b) Cohort study—For matched studies, give matching criteria and number of exposed and unexposed.  Case-control study—For matched studies, give matching criteria and the number of controls per case. | **nut-6** Report particular dietary, physiological or nutritional characteristics that were considered when selecting the target population. | 6 |
| Variables | 7 | Clearly define all outcomes, exposures, predictors, potential confounders, and effect modifiers. Give diagnostic criteria, if applicable. | **nut-7.1** Clearly define foods, food groups, nutrients, or other food components.  **nut-7.2** When using dietary patterns or indices, describe the methods to obtain them and their nutritional properties. | 7-9 |
| Data sources - measurements | 8 | For each variable of interest, give sources of data and details of methods of assessment (measurement).Describe comparability of assessment methods if there is more than one group. | **nut-8.1** Describe the dietary assessment method(s), e.g., portion size estimation, number of days and items recorded, how it was developed and administered, and how quality was assured. Report if and how supplement intake was assessed.  **nut-8.2** Describe and justify food composition data used. Explain the procedure to match food composition with consumption data. Describe the use of conversion factors, if applicable.  **nut-8.3** Describe the nutrient requirements, recommendations, or dietary guidelines and the evaluation approach used to compare intake with the dietary reference values, if applicable.  **nut-8.4** When using nutritional biomarkers, additionally use the STROBE Extension for Molecular Epidemiology (STROBE-ME). Report the type of biomarkers used and their usefulness as dietary exposure markers.  **nut-8.5** Describe the assessment of nondietary data (e.g., nutritional status and influencing factors) and timing of the assessment of these variables in relation to dietary assessment.  **nut-8.6** Report on the validity of the dietary or nutritional assessment methods and any internal or external validation used in the study, if applicable. | 10 |
| Bias | 9 | Describe any efforts to address potential sources of bias. | **nut-9** Report how bias in dietary or nutritional assessment was addressed, e.g., misreporting, changes in habits as a result of being measured, or data imputation from other sources | 10 |
| Study Size | 10 | Explain how the study size was arrived at. |  | **6** |
| Quantitative variables | 11 | Explain how quantitative variables were handled in the analyses. If applicable, describe which groupings were chosen and why. | **nut-11** Explain categorization of dietary/nutritional data (e.g., use of N-tiles and handling of nonconsumers) and the choice of reference category, if applicable. | 10 |
| Statistical  Methods | 12 | (a) Describe all statistical methods, including those used to control for confounding  (b) Describe any methods used to examine subgroups and interactions.  (c) Explain how missing data were addressed.  (d) Cohort study—If applicable, explain how loss to follow-up was addressed.  Case-control study—If applicable, explain how matching of cases and controls was addressed.  Cross-sectional study—If applicable, describe analytical methods taking account of sampling strategy.  (e) Describe any sensitivity analyses. | **nut-12.1** Describe any statistical method used to combine dietary or nutritional data, if applicable.  **nut-12.2** Describe and justify the method for energy adjustments, intake modeling, and use of weighting factors, if applicable.  **nut-12.3** Report any adjustments for measurement error, i.e,. from a validity or calibration study. | 10-11 |
| **Results** |  |  |  |  |
| Participants | 13 | (a) Report the numbers of individuals at each stage of the study—e.g., numbers potentially eligible, examined for eligibility, confirmed eligible, included in the study, completing follow-up, and analyzed.  (b) Give reasons for non-participation at each stage.  (c) Consider use of a flow diagram. | **nut-13** Report the number of individuals excluded based on missing, incomplete or implausible dietary/nutritional data. | 12 |
| Descriptive data | 14 | (a) Give characteristics of study participants (e.g., demographic, clinical, social) and information on exposures and potential confounders  (b) Indicate the number of participants with missing data for each variable of interest  (c) Cohort study—Summarize follow-up time (e.g., average and total amount) | **nut-14** Give the distribution of participant characteristics across the exposure variables if applicable. Specify if food consumption of total population or consumers only were used to obtain results. | 12 |
| Outcome data | 15 | Cohort study—Report numbers of outcome events or summary measures over time.  Case-control study—Report numbers in each exposure category, or summary measures of exposure.  Cross-sectional study—Report numbers of outcome events or summary measures. |  | 12 |
| Main results | 16 | (a) Give unadjusted estimates and, if applicable, confounder-adjusted estimates and their precision (e.g., 95% confidence interval).  Make clear which confounders were adjusted for and why they were included.  (b) Report category boundaries when continuous variables were categorized.  (c) If relevant, consider translating estimates of relative risk into absolute risk for a meaningful time period. | **nut-16** Specify if nutrient intakes are reported with or without inclusion of dietary supplement intake, if applicable. | 12-13 |
| Other analyses | 17 | Report other analyses done—e.g., analyses of subgroups and interactions and sensitivity analyses. | **nut-17** Report any sensitivity analysis (e.g., exclusion of misreporters or outliers) and data imputation, if applicable. | n/a |
| **Discussion** |  |  |  |  |
| Key results | 18 | Summarize key results with reference to study objectives. |  | 13-14 |
| Limitation | 19 | Discuss limitations of the study, taking into account sources of potential bias or imprecision. Discuss both direction and magnitude of any potential bias. | **nut-19** Describe the main limitations of the data sources and assessment methods used and implications for the interpretation of the findings. | 17-18 |
| Interpretation | 20 | Give a cautious overall interpretation of results considering objectives, limitations, multiplicity of analyses, results from similar studies, and other relevant evidence. | **nut-20** Report the nutritional relevance of the findings, given the complexity of diet or nutrition as an exposure. | 13-17 |
| Generalizability | 21 | Discuss the generalizability (external validity) of the study results. |  | 17 |
| **Other information** |  |  |  |  |
| Funding | 22 | Give the source of funding and the role of the funders for the present study and, if applicable, for the original study on which the present article is based. |  | 19 |
| *Ethics* |  |  | **nut-22.1** Describe the procedure for consent and study approval from ethics committee(s). | 20 |
| *Supplementary material* |  |  | **nut-22.2** Provide data collection tools and data as online material or explain how they can be accessed. | Supplementary materials file |

**Supplementary Table 2**: HEI-2015^1^ Components and Scoring Standards

| **No** | **Component** | **Maximum points** | **Standard for maximum score** | **Standard for minimum score of zero** |
| --- | --- | --- | --- | --- |
| **Adequacy:** |  |  |  |  |
|  | Total Fruits^2^ | 5 | ≥0.8 cup equivalent per 1,000 kcal | No Fruit |
|  | Whole Fruits^3^ | 5 | ≥0.4 cup equivalent per 1,000 kcal | No Whole Fruit |
|  | Total Vegetables^4^ | 5 | ≥1.1 cup equivalent per 1,000 kcal | No Vegetables |
|  | Greens and Beans^4^ | 5 | ≥0.2 cup equivalent per 1,000 kcal | No Dark-Green Vegetables or Legumes |
|  | Whole Grains | 10 | ≥1.5 ounce equivalent per 1,000 kcal | No Whole Grains |
|  | Dairy^5^ | 10 | ≥1.3 cup equivalent per 1,000 kcal | No Dairy |
|  | Total Protein Foods^4^ | 5 | ≥2.5 ounce equivalent per 1,000 kcal | No Protein Foods |
|  | Seafood and Plant Proteins^4,6^ | 5 | ≥0.8 ounce equivalent per 1,000 kcal | No Seafood or Plant Proteins |
|  | Fatty Acids^7^ | 10 | (PUFAs + MUFAs) / SFAs ≥2.5 | (PUFAs + MUFAs)/SFAs ≤1.2 |
| **Moderation** |  |  |  |  |
|  | Refined Grains | 10 | ≤1.8 ounce equivalent per 1,000 kcal | ≥4.3 ounce equivalent per 1,000 kcal |
|  | Sodium | 10 | ≤1.1 grams per 1,000 kcal | ≥2.0 grams per 1,000 kcal |
|  | Added Sugars | 10 | ≤6.5% of energy | ≥26% of energy |
|  | Saturated Fats | 10 | ≤8% of energy | ≥16% of energy |

Intakes between the minimum and maximum standards are scored proportionately.

^2^ Includes 100% fruit juice.

^3^ Includes all forms except juice.

^4^ Includes legumes (beans and peas).

^5^ Includes all milk products, such as fluid milk, yogurt, and cheese, and fortified soy beverages.

^6^ Includes seafood; nuts, seeds, soy products (other than beverages), and legumes (beans and peas).

^7^ Ratio of poly- and mono-unsaturated fatty acids (PUFAs and MUFAs) to saturated fatty acids (SFAs).

**Supplementary Table 3.** Scoring system and classification of food items in the NHANES^1^

| **Food groups** | **PDI** | **hPDI** | **uPDI** | **Pro-vegetarian** |
| --- | --- | --- | --- | --- |
| **Healthy plant foods** |  | | | |
| **Food group** | **Used in the calculation** | | | |
| Whole grains | Yes | Yes | Yes (reverse) | Yes^2^ |
| Fruits | Yes | Yes | Yes (reverse) | Yes |
| Vegetables | Yes | Yes | Yes (reverse) | Yes |
| Nuts | Yes | Yes | Yes (reverse) | Yes |
| Legumes | Yes | Yes | Yes (reverse) | Yes |
| Tea and coffee | Yes | Yes | Yes (reverse) | Not scored |
|  | | | | |
| Refined grains | Yes | Yes (reverse) | Yes | Yes^2^ |
| Potatoes | Yes | Yes (reverse) | Yes | Yes |
| Sugar sweetened beverages | Yes | Yes (reverse) | Yes | Not scored |
| Sweets and desserts | Yes | Yes (reverse) | Yes | Not scored |
| Salty food group | Yes | Yes (reverse) | Yes | Not scored |
| **Animal foods** |  | | | |
| Animal fat | Yes (reverse) | Yes (reverse) | Yes (reverse) | Yes (reverse) |
| Dairy | Yes (reverse) | Yes (reverse) | Yes (reverse) | Yes (reverse) |
| Eggs | Yes (reverse) | Yes (reverse) | Yes (reverse) | Yes (reverse) |
| Fish | Yes (reverse) | Yes (reverse) | Yes (reverse) | Yes (reverse) |
| Meat | Yes (reverse) | Yes (reverse) | Yes (reverse) | Yes (reverse) |
| Miscellaneous animal foods | Yes (reverse) | Yes (reverse) | Yes (reverse) | Not scored |

^1^ The PDI, hPDI, and uPDI categorized good groups to “healthy plant foods,” “less healthy plant foods,” and “animal foods.” The PVDI categorized food groups into “plant foods” and “animal foods.” Positive indicates that higher intakes received higher scores. Reverse indicates that higher intakes received lower scores.

^2^ Whole grains and refined grains were aggregated to “grains” food group in the PVDI.

PDI, overall plant-based diet index; hPDI, healthful plant-based diet index; uPDI, unhealthful plant-based diet index. PVDI, pro-vegetarian dietary index

**Supplementary Table 4.** Variables and method of assessment.

| **Variable name** | **Assessment methods** | **Categories/unit** |
| --- | --- | --- |
| **Socio-demographic** |  |  |
| Age | Self-reported age in years | years |
| Sex | Self-reported gender of the sample person | Male/female |
| Race | Self-reported race/ethnicity of response options: | Mexican American/ Other Hispanic/ Non-Hispanic White/ Non-Hispanic Black |
| Education | Self-reported |  |
| Marital status | Self-reported marital status | Married/living with partner/Widowed/Divorced/Separated/Never married |
| Income (Poverty-income ratio) | Self-reported family income, poverty measure derived from guidelines at time of assessment.  This variable is an index for the ratio of family income to poverty. The Department of Health and Human Services’ (HHS) poverty guidelines were used as the poverty measure to calculate this index (see variable INDFMPIR). | Ratio (continuous) |
| **Behavioural factors** |  |  |
| Smoking | Self-reported; Smoking status was determined as: never, former (smoked > 100 cigarettes in lifetime but does not currently smoke), and current (smoked > 100 cigarettes in lifetime and smokes currently). | Non-smoker, ex-smoker and smoker |
|  |  |  |
| Physical activity level | Self-reported; Levels of physical activity were assessed and scored using the validated Global Physical Activity Questionnaire Analysis (GPAQ (1). Specifically, the number of minutes that participants spent each week doing moderate to vigorous activities requiring at least 4 metabolic equivalent units (MET) per hour were estimated (2).  Insufficient (<600 MET-minute per week)/moderate ≥600 & <1200 MET-minute per week)/sufficient (≥1200 MET-minute per week). | Insufficient, moderate and sufficient |
| Alcohol intake | Self-reported; based on 24-hour dietary recall. | Continuous |
| High body mass index | Based on measured weight and height, BMI was computed (kg/m^2^). | Continuous |
| **Chronic conditions** |  |  |
| Cardiovascular disease (CVD) | Self-reported; CVD was a composite variable of any of the following five conditions:  Has a doctor or other health professional ever told you that you had   1. congestive heart failure? 2. coronary heart disease 3. angina/angina pectoris 4. heart attack (also called myocardial infarction) | Yes/no |
| Cancer | Self-reported; Have you ever been told by a doctor or other health professional that you had cancer or a malignancy of any kind? | Yes/no |
| Diabetes | Self-reported or measured (fasting glucose level ≥126 mg/dL or oral glucose tolerance test ≥ 2000 mg/dL or random glucose level ≥ 200 mg/dL or HbA1C ≥6.5% or having diabetes complications or insulin intake or self-reported doctor diagnosed diabetes) | Yes/no |
| Total cholesterol | Measured | Continuous |

MET *metabolic equivalent of task*. Information about questions, response options, and variable names for co-variates can be found here: <https://wwwn.cdc.gov/nchs/nhanes/default.aspx>

**Supplementary Table 5.** Characteristics of participants within tertiles of uPDI and hPDI.

| Covariates | uPDI | | | p-trend | hPDI | | | p-trend |
| --- | --- | --- | --- | --- | --- | --- | --- | --- |
|  | T1 | T2 | T3 |  | T1 | T2 | T3 |  |
| Age (years) (median, IQR) | 41 (30, 55) | 46 (33, 59) | 51 (1.8, 5) | <0.001 | 49 (36, 61) | 46 (33, 60) | 41 (29, 55) | <0.001 |
|  |  |  |  |  |  |  |  |  |
| Sex (n%) |  |  |  |  |  |  |  |  |
| Male | 59.8 | 49.4 | 42.3 | <0.001 | 53 | 49.9 | 48 | <0.001 |
| Female | 40.2 | 50.6 | 57.7 |  | 47 | 50.1 | 52 |  |
|  |  |  |  |  |  |  |  |  |
| Race (n%) |  |  |  |  |  |  |  |  |
| Mexican American | 8.2 | 8.4 | 6.7 | <0.001 | 5.7 | 8 | 10.4 | <0.001 |
| Other Hispanic | 5.1 | 4.7 | 4.8 |  | 3.7 | 4.7 | 6.6 |  |
| Non-Hispanic White | 69.2 | 72.1 | 73.4 |  | 79.1 | 70.7 | 62 |  |
| Non-Hispanic Black | 12.6 | 9.5 | 6.7 |  | 5.4 | 10.1 | 14.9 |  |
| Other Race - Including Multi-Racial | 4.9 | 5.4 | 8.5 |  | 6.2 | 6.5 | 6.2 |  |
|  |  |  |  |  |  |  |  |  |
| Marital Status (n%) |  |  |  |  |  |  |  |  |
| Married/living with partner | 62.7 | 62.9 | 66.9 | <0.001 | 69 | 64.3 | 57.4 | <0.001 |
| Widowed | 4.3 | 6.1 | 7.5 |  | 5.8 | 6.6 | 5.4 |  |
| Divorced | 9.6 | 10.4 | 10 |  | 9.5 | 10 | 10.7 |  |
| Separated | 2.6 | 2.8 | 1.9 |  | 1.9 | 2.3 | 3.3 |  |
| Never married | 20.8 | 17.8 | 13.7 |  | 13.7 | 16.7 | 23.2 |  |
|  |  |  |  |  |  |  |  |  |
| Education (n%) |  |  |  |  |  |  |  |  |
| Less Than High School | 17.1 | 17.1 | 17.3 | <0.001 | 10.1 | 10.1 | 16.6 | <0.001 |
| High School Diploma (including GED) | 28.7 | 28.7 | 24.2 |  | 20.9 | 20.9 | 25.9 |  |
| More Than High School | 54.2 | 54.2 | 58.5 |  | 69 | 69 | 57.5 |  |
|  |  |  |  |  |  |  |  |  |
| FPIR (median, IQR) | 2.9 (1.5, 4.8) | 3.1 (1.5, 5) | 3.4 (1.8, 5) | <0.001 | 3.9 (2.1, 5) | 3.0 (1.6, 5) | 2.3 (1.2, 4.2) | <0.001 |
|  |  |  |  |  |  |  |  |  |
| Smoking (n%) |  |  |  |  |  |  |  |  |
| Never smoked | 50.5 | 52.4 | 56.1 | <0.001 | 54.5 | 52.3 | 51.6 | <0.001 |
| Ex-smoker | 22.9 | 25 | 28.2 |  | 29.7 | 25.6 | 19.1 |  |
| Smoker | 26.7 | 22.6 | 15.8 |  | 15.8 | 22.1 | 29.3 |  |
|  |  |  |  |  |  |  |  |  |
| Physical Activity Level (n%) |  |  |  |  |  |  |  |  |
| Low | 36.3 | 35.7 | 34.2 | 0.2 | 30.2 | 37 | 40.7 | <0.001 |
| Moderate | 12.2 | 13.2 | 13.2 |  | 13.9 | 12.4 | 12 |  |
| High | 51.5 | 51.1 | 52.6 |  | 55.9 | 50.6 | 47.3 |  |
|  |  |  |  |  |  |  |  |  |
| Alcohol intake (gm) (median, IQR) | 0 (0, 4.7) | 0 (0, 7.3) | 0 (0, 1.1) | <0.001 | 0 (0, 12.4) | 0 (0, 0.5) | 0 (0, 0) | 0.3 |
|  |  |  |  |  |  |  |  |  |
| CVD (n%) |  |  |  |  |  |  |  |  |
| No | 93 | 91.1 | 90.5 | <0.001 | 91.2 | 91.6 | 91.5 | 0.48 |
| Yes | 7 | 8.9 | 9.5 |  | 8.2 | 8.8 | 8.4 |  |
|  |  |  |  |  |  |  |  |  |
| Cancer (n%) |  |  |  |  |  |  |  |  |
| No | 92.4 | 90.8 | 88.8 | 0.001 | 88.9 | 91.1 | 92.6 | 0.001 |
| Yes | 7.6 | 9.2 | 11.2 |  | 11.1 | 8.9 | 7.4 |  |
|  |  |  |  |  |  |  |  |  |
| Hypertension (n%) |  |  |  |  |  |  |  |  |
| No | 72.3 | 68.9 | 64.9 | <0.001 | 67.9 | 67.7 | 71 | 0.004 |
| Yes | 27.7 | 31.1 | 35.1 |  | 32.1 | 32.3 | 29 |  |
|  |  |  |  |  |  |  |  |  |
| Arthritis (n%) |  |  |  |  |  |  |  |  |
| No | 78.5 | 74.9 | 72.5 | <0.001 | 74.2 | 74.6 | 77.8 | <0.001 |
| Yes | 21.5 | 25.1 | 27.5 |  | 25.8 | 25.4 | 22.2 |  |
|  |  |  |  |  |  |  |  |  |
| Diabetes^n^(n%) |  |  |  |  |  |  |  |  |
| No | 90.5 | 88.6 | 86.9 | <0.001 | 88.2 | 88.9 | 89.1 | 0.42 |
| Yes | 9.5 | 11.4 | 13.1 |  | 11.8 | 11.1 | 10.9 |  |
|  |  |  |  |  |  |  |  |  |
| Cholesterol (mean, SD) | 5.1 (1.1) | 5.1 (1.1) | 5.2 (1.1) | 0.001 | 5.1 (1.0) | 5.1 (1.1) | 5.1 (1.2) | 0.01 |
|  |  |  |  |  |  |  |  |  |
| BMI (mean, SD) | 28.5 (6.3) | 28.2 (6.1) | 27.9 (5.8) | <0.001 | 28.1 (5.5) | 28.1 (6.2) | 28.3 (6.8) | 0.045 |
|  |  |  |  |  |  |  |  |  |
| Obesity (n%) |  |  |  |  |  |  |  |  |
| No | 64.9 | 66.6 | 69.6 | <0.001 | 68 | 68 | 64.6 | 0.002 |
| Yes | 35.1 | 33.4 | 30.4 |  | 32 | 32 | 35.4 |  |
|  |  |  |  |  |  |  |  |  |
| CRP (n%) |  |  |  |  |  |  |  |  |
| < 1.0 mg/L | 33.3 | 33.9 | 36 | 0.02 | 36.4 | 34.1 | 32 | <0.001 |
| 1.0-3.0 mg/L | 37.2 | 38.1 | 37.3 |  | 38.5 | 36.9 | 37 |  |
| > 3.0 mg/L | 29.5 | 28 | 26.6 |  | 25.1 | 29 | 31.1 |  |
|  |  |  |  |  |  |  |  |  |

*HEI-2015* Healthy Eating Index 2015, *PDI* Plant-based dietaty index, *PVD* Provegetarian diet, *CRP* C-reactive protein, *FPIR* Family poverty income ratio, CVD cardiovascular diseases, *BMI* Body mass index.

ANOVA was used for quantitative variables and Pearson’s chi-square test was used for categorical variables.

**Supplementary Table 6.** Cross-sectional association between HEI-2015 and systemic inflammation in NHANES 1999-2010 and 2015-2018.

| Model | Odds Ratio (95% Confidence Interval) | | | p-trend |
| --- | --- | --- | --- | --- |
|  | T1 | T2 | T3 |  |
| All population | | | | |
| Model 1 | 1.00 | 0.80(0.73-0.88) | 0.64(0.58-0.70) | **<0.001** |
| Model 2 | 1.00 | 0.82(0.74-0.90) | 0.68(0.62-0.74) | **<0.001** |
| Model 3 | 1.00 | 0.83(0.75-0.91) | 0.68(0.62-0.75) | **<0.001** |
| Model 4 | 1.00 | 0.84(0.75-0.93) | 0.76(0.69-0.84) | **<0.001** |
|  |  |  |  |  |
| Obesity | | | | |
| Model 1 | 1.00 | 0.80(0.69-0.94) | 0.72(0.62-0.83) | **<0.001** |
| Model 2 | 1.00 | 0.81(0.69-0.95) | 0.75(0.64-0.87) | **<0.001** |
| Model 3 | 1.00 | 0.81(0.69-0.95) | 0.75(0.64-0.87) | **<0.001** |
|  |  |  |  |  |
| Non-obesity | | | | |
| Model 1 | 1.00 | 0.84(0.73-0.96) | 0.71(0.63-0.81) | **<0.001** |
| Model 2 | 1.00 | 0.87(0.75-1.00) | 0.77(0.68-0.89) | **<0.001** |
| Model 3 | 1.00 | 0.87(0.75-1.00) | 0.78(0.69-0.89) | **<0.001** |

Odds ratio from multivariable logistic regression. Total participants (N=27,915), non-obesity (N=18,454), obesity (N=9,461).

Model 1: adjusted for sex, age, race, marital status, education, family poverty to income ratio

Model 2: additionally adjusted for smoking, physical activity, and alcohol intake

Model 3: additionally adjusted for cardiovascular disease, cancer, hypertension, arthritis, diabetes and cholesterol

Model 4: additionally adjusted for BMI

Bold indicates significant in p-value

**Supplementary Table 7.** Cross-sectional association between plant-based dietary indices and systemic inflammation in NHANES 1999-2010 and 2015-2018.

| Model | Odds Ratio (95% Confidence Interval) | | | p-trend |
| --- | --- | --- | --- | --- |
|  | T1 | T2 | T3 |  |
| PDI |  |  |  |  |
| All population |  |  |  |  |
| Model 1 | 1.00 | 0.82(0.76-0.89) | 0.71(0.66-0.78) | **<0.001** |
| Model 2 | 1.00 | 0.82(0.76-0.89) | 0.72(0.67-0.78) | **<0.001** |
| Model 3 | 1.00 | 0.83(0.76-0.90) | 0.74(0.68-0.81) | **<0.001** |
| Model 4 | 1.00 | 0.87(0.79-0.95) | 0.83(0.75-0.91) | **<0.001** |
|  |  |  |  |  |
| Obesity |  |  |  |  |
| Model 1 | 1.00 | 0.86(0.74-1.00) | 0.78(0.67-0.91) | **0.002** |
| Model 2 | 1.00 | 0.85(0.73-0.99) | 0.78(0.67-0.91) | **0.002** |
| Model 3 | 1.00 | 0.85(0.73-0.99) | 0.78(0.67-0.92) | **0.002** |
|  |  |  |  |  |
| Non-obesity |  |  |  |  |
| Model 1 | 1.00 | 0.82(0.73-0.93) | 0.75(0.67-0.84) | **<0.001** |
| Model 2 | 1.00 | 0.84(0.75-0.95) | 0.78(0.70-0.88) | **<0.001** |
| Model 3 | 1.00 | 0.86(0.76-0.97) | 0.82(0.73-0.92) | **0.001** |
| hPDI |  |  |  |  |
| All population |  |  |  |  |
| Model 1 | 1.00 | 0.86(0.78-0.94) | 0.77(0.70-0.85) | **<0.001** |
| Model 2 | 1.00 | 0.87(0.79-0.95) | 0.79(0.71-0.87) | **<0.001** |
| Model 3 | 1.00 | 0.87(0.79-0.96) | 0.79(0.71-0.87) | **<0.001** |
| Model 4 | 1.00 | 0.85(0.77-0.95) | 0.79(0.71-0.88) | **<0.001** |
|  |  |  |  |  |
| Obesity |  |  |  |  |
| Model 1 | 1.00 | 0.90(0.77-1.05) | 0.82(0.70-0.97) | **0.02** |
| Model 2 | 1.00 | 0.90(0.76-1.05) | 0.82(0.70-0.97) | **0.02** |
| Model 3 | 1.00 | 0.89(0.75-1.05) | 0.80(0.67-0.96) | **0.02** |
|  |  |  |  |  |
| Non-obesity |  |  |  |  |
| Model 1 | 1.00 | 0.92(0.82-1.04) | 0.79(0.70-0.90) | **<0.001** |
| Model 2 | 1.00 | 0.94(0.83-1.06) | 0.83(0.73-0.94) | **0.004** |
| Model 3 | 1.00 | 0.89(0.79-1.00) | 0.77(0.67-0.88) | **<0.001** |
| uPDI |  |  |  |  |
| All population |  |  |  |  |
| Model 1 | 1.00 | 1.16(1.06-1.28) | 1.26(1.15-1.39) | **<0.001** |
| Model 2 | 1.00 | 1.14(1.04-1.25) | 1.22(1.11-1.35) | **<0.001** |
| Model 3 | 1.00 | 1.14(1.03-1.25) | 1.21(1.10-1.34) | **<0.001** |
| Model 4 | 1.00 | 1.15(1.04-1.28) | 1.18(1.06-1.31) | **0.002** |
|  |  |  |  |  |
| Obesity |  |  |  |  |
| Model 1 | 1.00 | 1.10(0.95-1.27) | 1.21(1.04-1.40) | **0.01** |
| Model 2 | 1.00 | 1.09(0.94-1.27) | 1.19(1.03-1.38) | **0.02** |
| Model 3 | 1.00 | 1.09(0.94-1.27) | 1.21(1.04-1.42) | **0.02** |
|  |  |  |  |  |
| Non-obesity |  |  |  |  |
| Model 1 | 1.00 | 1.25(1.12-1.40) | 1.26(1.11-1.42) | **<0.001** |
| Model 2 | 1.00 | 1.22(1.08-1.36) | 1.19(1.05-1.35) | **0.003** |
| Model 3 | 1.00 | 1.20(1.07-1.34) | 1.14(1.00-1.29) | **0.03** |

Odds ratio from multivariable logistic regression. Total participants (N=27,915), non-obesity (N=18,454), obesity (N=9,461).

Model 1: adjusted for sex, age, race, marital status, education, family poverty to income ratio

Model 2: additionally adjusted for smoking, physical activity, and alcohol intake

Model 3: additionally adjusted for cardiovascular disease, cancer, hypertension, arthritis, diabetes and cholesterol

Model 4: additionally adjusted for BMI

Bold indicates significant in p-value

**Supplementary Table 8.** Cross-sectional association between pro-vegetarian diet and systemic inflammation in NHANES 1999-2010 and 2015-2018.

| Model | Odds Ratio (95% Confidence Interval) | | | p-trend |
| --- | --- | --- | --- | --- |
|  | T1 | T2 | T3 |  |
| All population |  |  |  |  |
| Model 1 | 1.00 | 0.83(0.77-0.91) | 0.72(0.65-0.79) | **<0.001** |
| Model 2 | 1.00 | 0.84(0.78-0.92) | 0.74(0.67-0.81) | **<0.001** |
| Model 3 | 1.00 | 0.85(0.78-0.92) | 0.76(0.69-0.83) | **<0.001** |
| Model 4 | 1.00 | 0.88(0.81-0.96) | 0.85(0.76-0.95) | **0.003** |
|  |  |  |  |  |
| Obesity |  |  |  |  |
| Model 1 | 1.00 | 0.93(0.80-1.07) | 0.83(0.71-0.97) | **0.02** |
| Model 2 | 1.00 | 0.93(0.81-1.08) | 0.84(0.72-0.98) | **0.03** |
| Model 3 | 1.00 | 0.93(0.80-1.08) | 0.84(0.72-0.98) | **0.03** |
|  |  |  |  |  |
| Non-obesity |  |  |  |  |
| Model 1 | 1.00 | 0.81(0.71-0.91) | 0.77(0.68-0.88) | **<0.001** |
| Model 2 | 1.00 | 0.83(0.73-0.93) | 0.82(0.72-0.93) | **0.002** |
| Model 3 | 1.00 | 0.84(0.74-0.95) | 0.85(0.75-0.97) | **0.02** |

Odds ratio from multivariable logistic regression. Total participants (N=27,915), non-obesity (N=18,454), obesity (N=9,461).

Model 1: adjusted for sex, age, race, marital status, education, family poverty to income ratio

Model 2: additionally adjusted for smoking, physical activity, and alcohol intake

Model 3: additionally adjusted for cardiovascular disease, cancer, hypertension, arthritis, diabetes and cholesterol

Model 4: additionally adjusted for BMI

Bold indicates significant in p-value

**Supplementary Table 9.** Hazard ratio association between PDI, PVD and all-cause mortality risk in NHANES 1999-2008

| Model | Hazard Ratio (95% Confidence Interval) | | | p-trend |
| --- | --- | --- | --- | --- |
|  | T1 | T2 | T3 |  |
| PDI | | | | |
| Model 1 | 1.00 | 0.95(0.77-1.17) | 0.87(0.73-1.03) | 0.11 |
| Model 2 | 1.00 | 0.97(0.78-1.20) | 0.92(0.78-1.09) | 0.35 |
| Model 3 | 1.00 | 0.97(0.78-1.20) | 0.91(0.77-1.08) | 0.29 |
| Model 4 | 1.00 | 0.97(0.78-1.20) | 0.90(0.75-1.09) | 0.29 |
|  |  |  |  |  |
| hPDI | | | | |
| Model 1 | 1.00 | 1.02(0.79-1.30) | 1.01(0.79-1.28) | 0.96 |
| Model 2 | 1.00 | 1.01(0.79-1.29) | 1.04(0.82-1.32) | 0.73 |
| Model 3 | 1.00 | 1.02(0.79-1.30) | 1.03(0.81-1.32) | 0.79 |
| Model 4 | 1.00 | 1.03(0.79-1.34) | 1.05(0.80-1.38) | 0.73 |
|  |  |  |  |  |
| uPDI | | | | |
| Model 1 | 1.00 | 1.12(0.91-1.37) | 1.13(0.90-1.43) | 0.27 |
| Model 2 | 1.00 | 1.08(0.88-1.32) | 1.06(0.84-1.34) | 0.61 |
| Model 3 | 1.00 | 1.08(0.88-1.32) | 1.05(0.83-1.33) | 0.63 |
| Model 4 | 1.00 | 1.08(0.89-1.31) | 1.08(0.84-1.37) | 0.53 |
|  |  |  |  |  |
| PVD |  |  |  |  |
| Model 1 | 1.00 | 1.15(0.95-1.38) | 1.00(0.83-1.21) | 0.97 |
| Model 2 | 1.00 | 1.19(0.99-1.44) | 1.09(0.90-1.32) | 0.37 |
| Model 3 | 1.00 | 1.19(0.99-1.43) | 1.08(0.89-1.30) | 0.43 |
| Model 4 | 1.00 | 1.18(0.99-1.42) | 1.07(0.88-1.30) | 0.49 |

Hazard ratio from multivariable Cox proportional hazards. Total participants (N=11,939).

Model 1: adjusted for sex, age, race, marital status, education, family poverty to income ratio

Model 2: additionally adjusted for smoking, physical activity, and alcohol intake

Model 3: additionally adjusted for cardiovascular disease, cancer, hypertension, arthritis, diabetes and cholesterol

Model 4: additionally adjusted for BMI

Bold indicates significant in p-value

**Supplementary Table 10.** Joint associations of HEI-2015, hs-CRP with all-cause, CVD and cancer mortality risks in NHANES 1999-2008.

|  |  | HEI-2015 | | | | | | | | | | |
| --- | --- | --- | --- | --- | --- | --- | --- | --- | --- | --- | --- | --- |
|  |  | T1 | | |  | T2 | | |  | T3 | | |
|  |  | HR | 95% CI | |  | HR | 95% CI | |  | HR | 95% CI | |
|  | All-cause |  |  | |  |  |  | |  |  |  | |
|  | All population |  |  | |  |  |  | |  |  |  | |
| hs-CRP | Low | 1.37 | 0.88 | 2.14 |  | 0.91 | 0.65 | 1.29 |  | 1 | Ref | |
|  | Moderate | 1.28 | 0.86 | 1.90 |  | 0.93 | 0.66 | 1.31 |  | 1.09 | 0.81 | 1.45 |
|  | Severe | 1.26 | 0.86 | 1.83 |  | 1.45 | 1.02 | 2.05 |  | 1.31 | 0.95 | 1.81 |
|  |  |  |  |  |  |  |  |  |  |  |  |  |
|  | Obesity |  |  |  |  |  |  |  |  |  |  |  |
|  | Low | 2.17 | 0.69 | 6.79 |  | 0.27 | 0.07 | 1.06 |  | 1 | Ref | |
|  | Moderate | 1.73 | 0.66 | 4.52 |  | 0.85 | 0.35 | 2.06 |  | 1.45 | 0.61 | 3.44 |
|  | Severe | 1.27 | 0.49 | 3.33 |  | 1.43 | 0.63 | 3.20 |  | 1.57 | 0.69 | 3.59 |
|  |  |  |  |  |  |  |  |  |  |  |  |  |
|  | Non-obesity |  |  |  |  |  |  |  |  |  |  |  |
|  | Low | 1.26 | 0.74 | 2.15 |  | 1.05 | 0.72 | 1.51 |  | 1 | Ref | |
|  | Moderate | 1.11 | 0.70 | 1.75 |  | 0.97 | 0.66 | 1.42 |  | 1.01 | 0.73 | 1.40 |
|  | Severe | 1.11 | 0.71 | 1.72 |  | 1.36 | 0.90 | 2.05 |  | 1.26 | 0.87 | 1.82 |
|  |  |  |  |  |  |  |  |  |  |  |  |  |
|  | CVD |  |  |  |  |  |  |  |  |  |  |  |
|  | Low | 1.33 | 0.52 | 3.37 |  | 0.87 | 0.38 | 2.01 |  | 1.00 | Ref | |
|  | Moderate | 1.23 | 0.54 | 2.80 |  | 0.90 | 0.41 | 1.98 |  | 0.79 | 0.37 | 1.70 |
|  | Severe | 0.74 | 0.29 | 1.92 |  | 0.94 | 0.41 | 2.18 |  | 0.79 | 0.35 | 1.82 |
|  |  |  |  |  |  |  |  |  |  |  |  |  |
|  | Cancer |  |  |  |  |  |  |  |  |  |  |  |
|  | Low | 2.15 | 1.25 | 3.70 |  | 0.80 | 0.40 | 1.58 |  | 1.00 | Ref | |
|  | Moderate | 1.69 | 0.84 | 3.39 |  | 1.02 | 0.52 | 1.99 |  | 1.21 | 0.69 | 2.12 |
|  | Severe | 0.97 | 0.47 | 1.97 |  | 1.16 | 0.64 | 2.09 |  | 0.94 | 0.44 | 2.00 |

Hazard ratio from multivariable Cox proportional hazards. Total participants (N=11,939), non-obesity (N=8,596), obesity (N=3,343).

**Supplementary Figure 1.** A directed acyclic diagram of the relationship between diet, inflammation, obesity and mortality


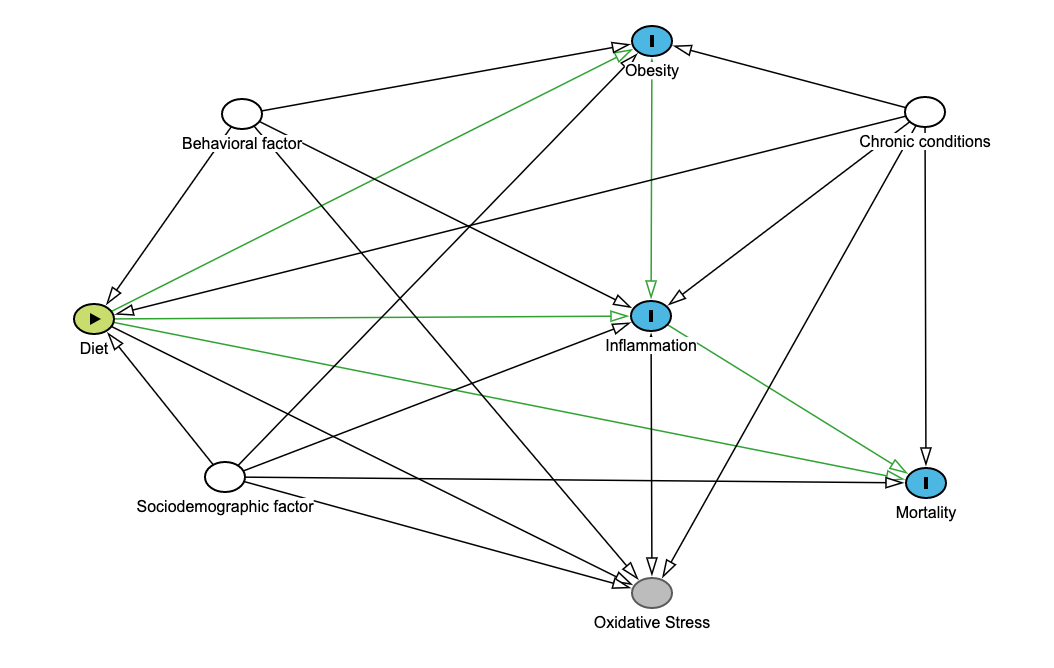


**Supplementary Figure 2.** Kaplan-Meier curves for HEI-2015 and CRP.


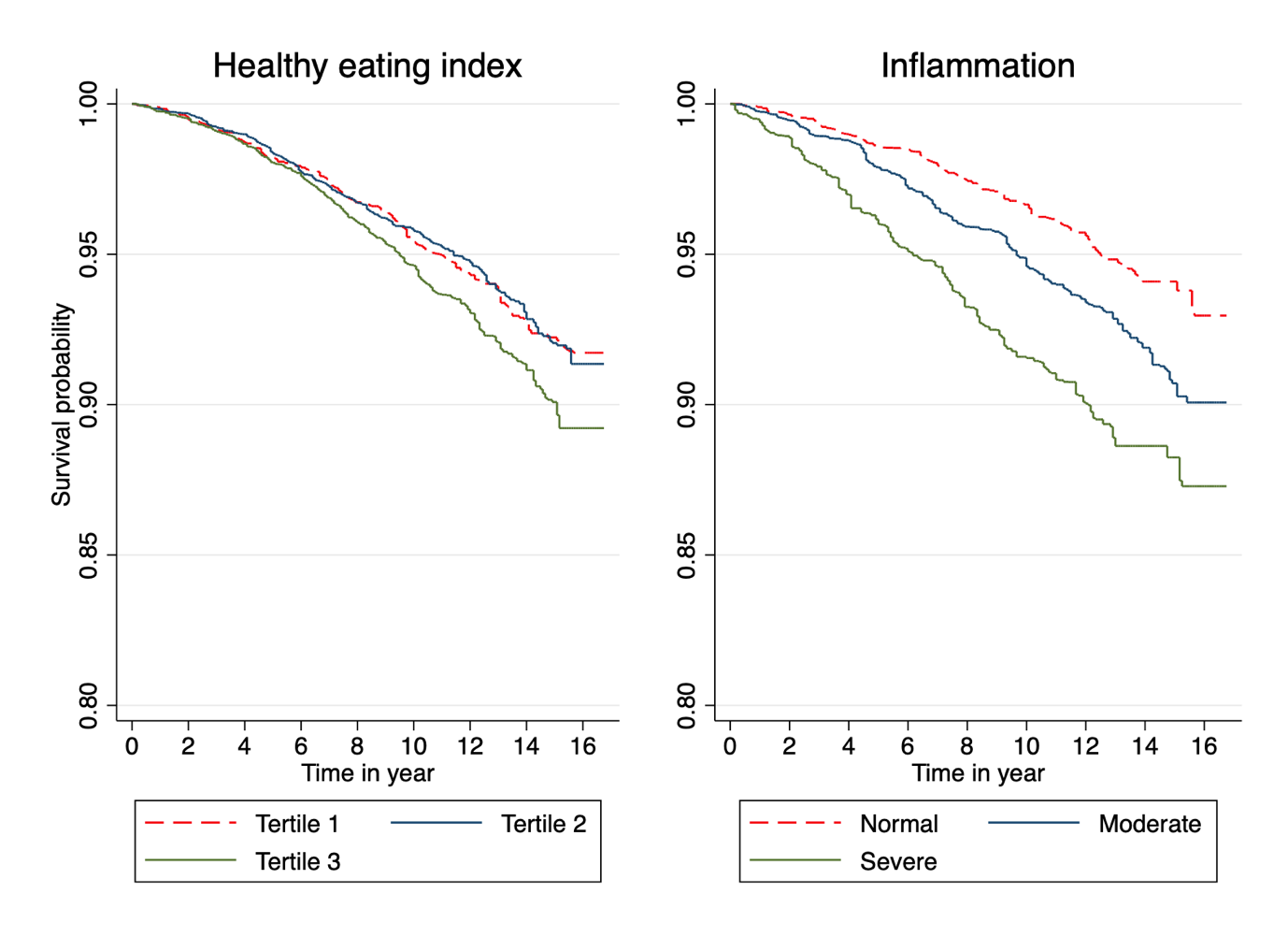

Supplement: Supplementary file 1 — Supplementary file1 (DOCX 5580 KB) [file 394_2023_3191_MOESM1_ESM.docx]
